# Supplementary material for: Strain-level characterization of broad host range mobile genetic elements transferring antibiotic resistance from the human microbiome
Source: Nat Commun. 2022 Mar 17;13:1445. doi: 10.1038/s41467-022-29096-9 (PMC8931123; doi:10.1038/s41467-022-29096-9)
Supplement: Supplementary file 1 — Supplementary Information [file 41467_2022_29096_MOESM1_ESM.pdf]

Supplementary Information: Strain-level characterization of broad host range mobile genetic elements transferring antibiotic resistance from the human microbiome

T. Lawley et al.

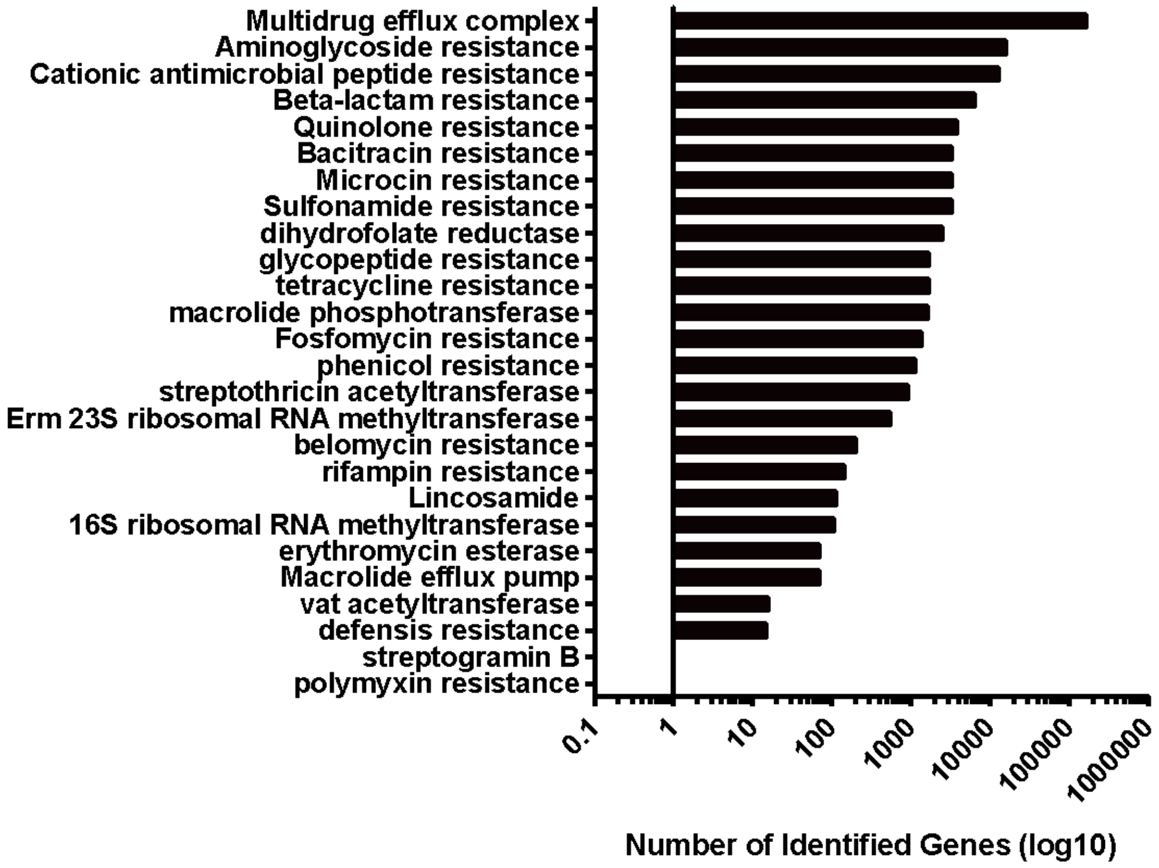

Supplementary Figure 1: ARG classes identified as shared between the pathogenic and symbiont bacterial genomes. Source data are provided as a Source Data file.

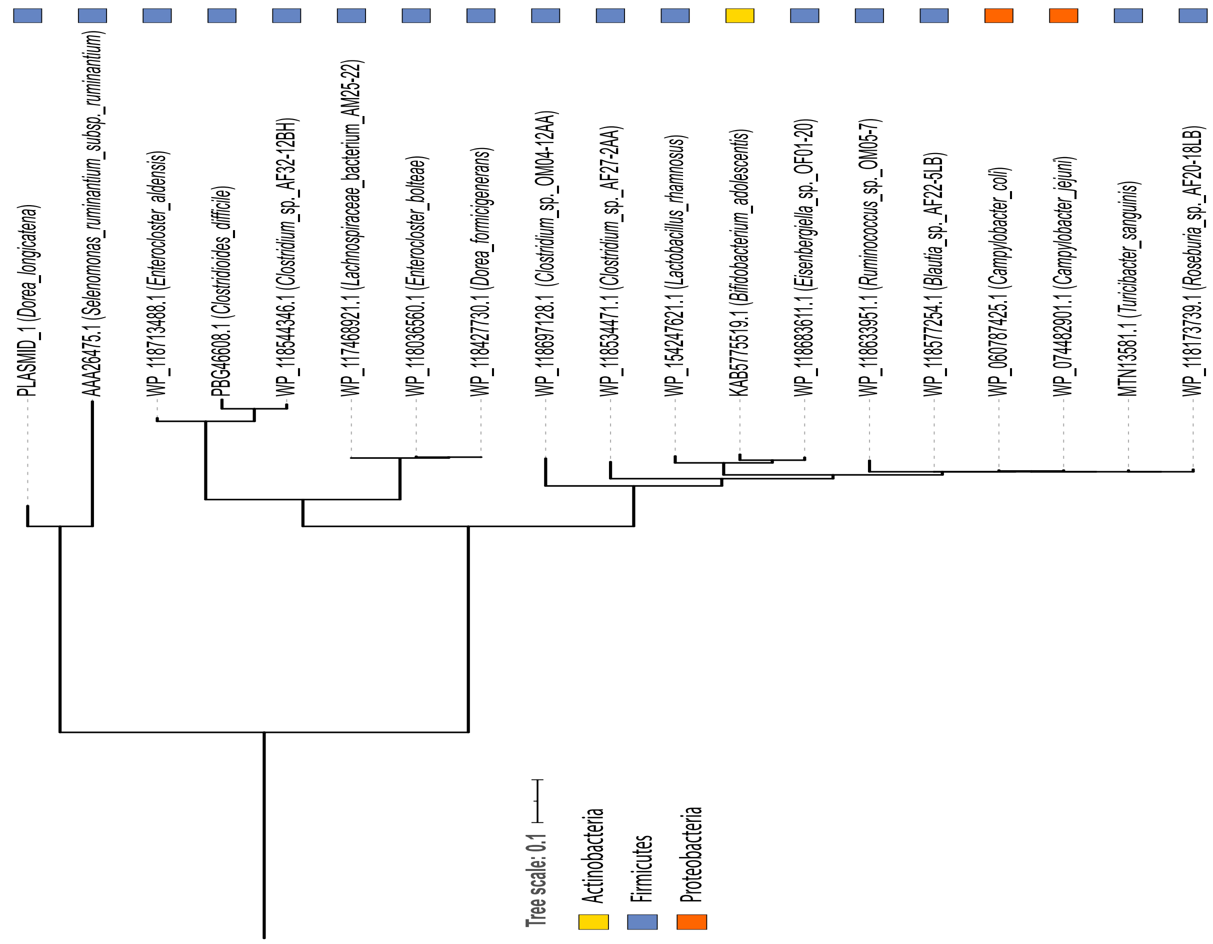

Supplementary Figure 2: Maximum likelihood Tree of RepA from PLASMID_1. Maximum likelihood tree of RepA from PLASMID_1 and 17 closely related homologues (14 Firmicutes (Blue); 2 Proteobacteria (Red); 1 Actinobacteria (Yellow).

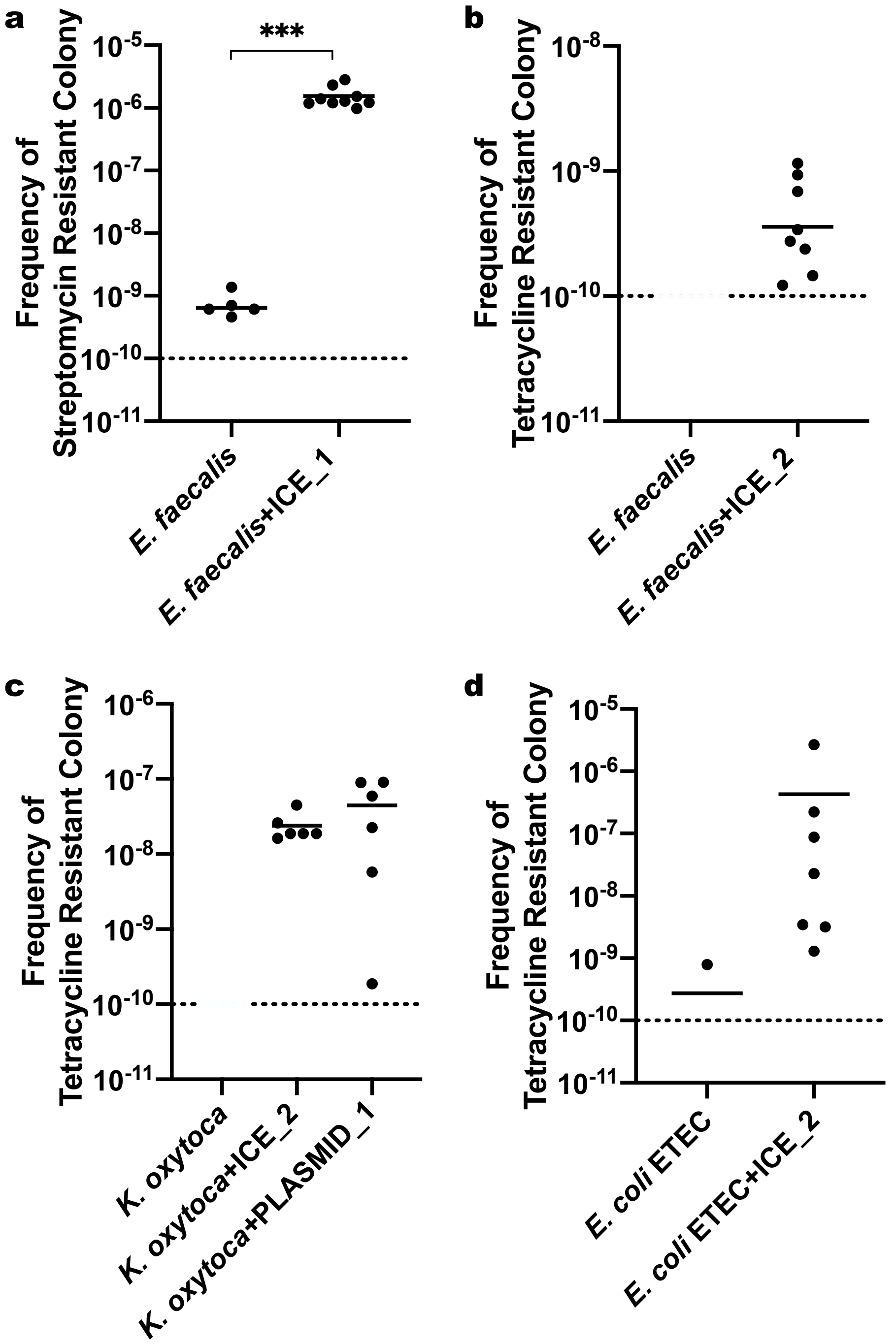

Supplementary Figure 3: Conjugation frequency of *E. faecalis, K. oxytoca* and *E. coli* ETEC (transconjugants per recipient). a, Streptomycin resistance arisen from spontaneous mutation in the recipient subjected to the same conjugation protocol without the donor was about 1,000 times lower than that of the conjugation (Two-tailed Mann-Whitney test, ****P*-value = 0.0004; n = 6, 9). b, Tetracycline resistance arising from spontaneous mutation was below detection limit 10^{-10} (n = 12, 12; high selection). c, The rate of spontaneous tetracycline resistant mutation was below detection limit 10^{-10} (n = 12, 6, 6; low selection). d, The rate of spontaneous tetracycline resistant mutation was below detection limit 10^{-10} except in one control experiment (n = 4, 7; low selection) The mean of each data set is indicated by a short horizontal line. Data points below detection limit (dashed line) are not shown. Source data are provided as a Source Data file.

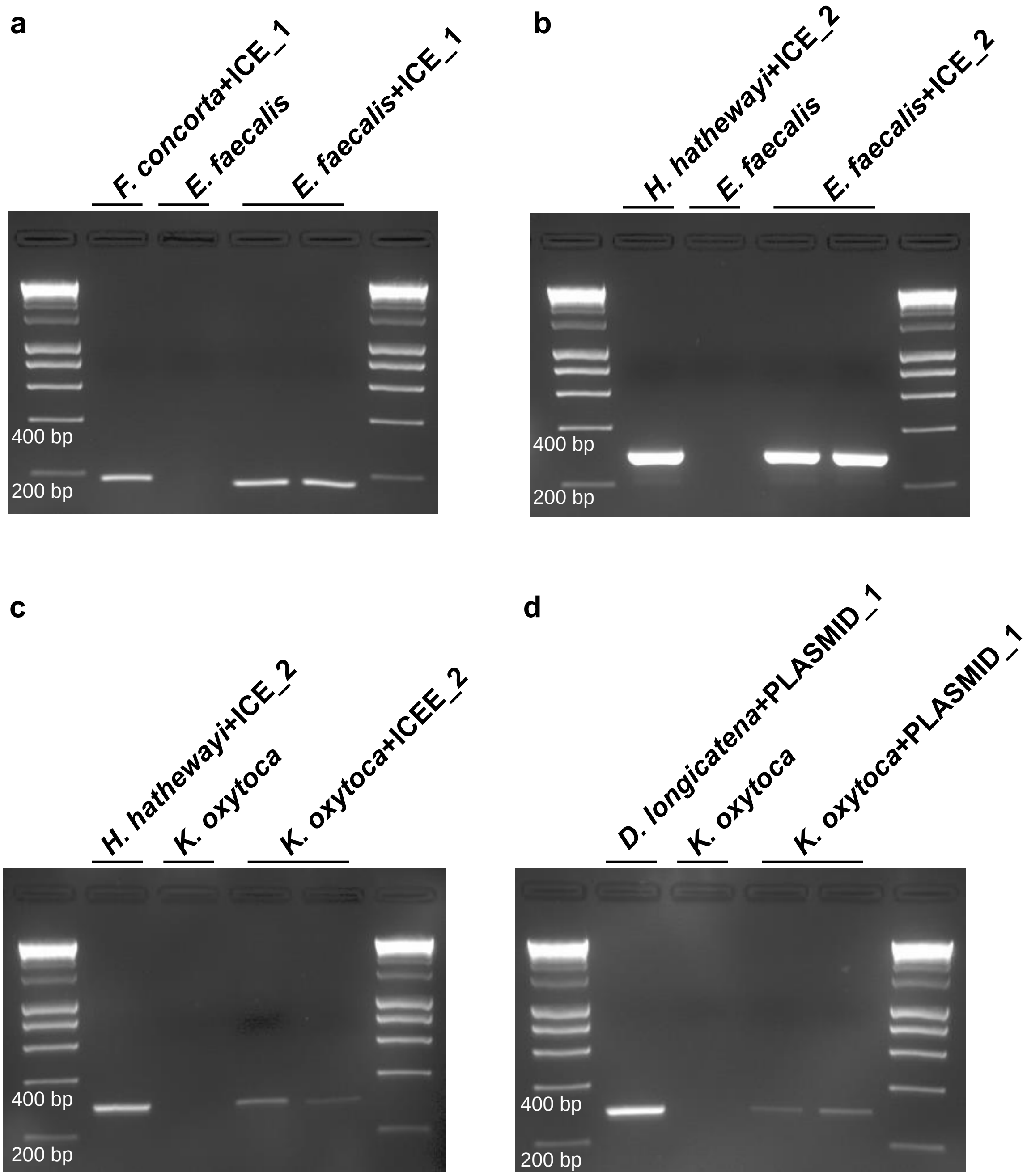

Supplementary Figure 4: Agarose gel (2%) electrophoresis of PCR products from all conjugations. a, conjugation of ICE_1 (product size 181 bp) from *F. contorta* into *E. faecalis* (n = 10); conjugation of ICE_2 (product size 294 bp) from *H. hathewayi* b**,** into *E. faecalis* (n = 23) and c, into *K. oxytoca* (n = 7); d, conjugation of PLASMID_1 (product size 301 bp) from *D. longicatena* into *K. oxytoca* (n = 4). Two transconjugants were selected for each pair of conjugation. HyperLadder™ 1 kb (Bioline; BIO-33026) used.

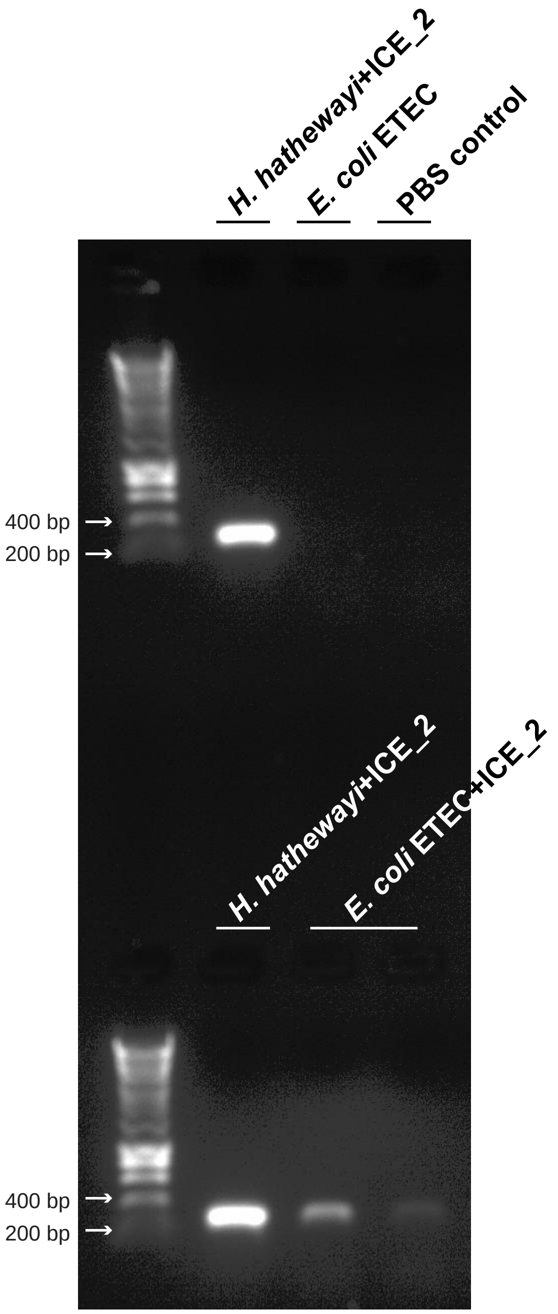

Supplementary Figure 5: Agarose gel (1%) electrophoresis of PCR product from conjugation of ICE_2 from *H. hathewayi* into *E. coli* ETEC. Product size is 294 bp. Two transconjugants were show here as representation (n = 42). HyperLadder™ 1 kb (Bioline; BIO-33026) was used.
