# Supplementary material for: Strain-level characterization of broad host range mobile genetic elements transferring antibiotic resistance from the human microbiome
Source: Nat Commun. 2022 Mar 17;13:1445. doi: 10.1038/s41467-022-29096-9 (PMC8931123; doi:10.1038/s41467-022-29096-9)
Supplement: Supplementary file 3 — Description of Additional Supplementary Files [file 41467_2022_29096_MOESM3_ESM.docx]

**Description of Additional Supplementary Files**

**File Name:** Supplementary Data 1

**Description:** List of 1354 isolates from the HGG collection

**File Name:** Supplementary Data 2

**Description:** List of 45403 pathogen isolates

**File Name:** Supplementary Data 3

**Description:** Summary of 15 broad host range MGE features

**File Name:** Supplementary Data 4

**Description:** Comparison of RepA in identified MGEs

**File Name:** Supplementary Data 5

**Description:** Donor, recipient and transconjugant strains sequenced with long-read sequencing

**File Name:** Supplementary Data 6

**Description:** Genebank files (concatenated) of 15 broad host range MGEs
